# Inhibition of TRIB3 Protects Against Neurotoxic Injury Induced by Kainic Acid in Rats

**DOI:** 10.3389/fphar.2019.00585

**Published:** 2019-05-22

**Authors:** Jing Zhang, Ying Han, Yang Zhao, Qinrui Li, Hongfang Jin, Jiong Qin

**Affiliations:** ^1^ Department of Pediatrics, Peking University First Hospital, Beijing, China; ^2^ Department of Pediatrics, Peking University People’s Hospital, Beijing, China

**Keywords:** epilepsy, endoplasmic reticulum stress, tribbles pseudokinase 3, neuronal apoptosis, kainic acid

## Abstract

Epilepsy refers to a group of neurological disorders of varying etiologies characterized by recurrent seizures, resulting in brain dysfunction. Endoplasmic reticulum (ER) stress is highly activated in the process of epilepsy-related brain injury. However, the mechanisms by which ER stress triggers neuronal apoptosis remain to be fully elucidated. Tribbles pseudokinase 3 (TRIB3) is a pseudokinase that affects a number of cellular functions, and its expression is increased during ER stress. Here, we sought to clarify the role of TRIB3 in neuronal apoptosis mediated by ER stress. In the kainic acid (KA) (10 mg/kg)-induced rat seizure model, we characterized neuronal injury and apoptosis after KA injection. KA induced an ER stress response, as indicated by elevated expression of glucose-regulated protein 78 (GRP78) and C/EBP homologous protein (CHOP). TRIB3 protein was upregulated concomitantly with the downregulation of phosphorylated-protein kinase B (p-AKT) in rats following KA administration. In rat cortical neurons treated with KA, TRIB3 knockdown by siRNA reduced the number of dying neurons, decreased the induction of GRP78 and CHOP and the activation of caspase-3, and blocked the dephosphorylation of AKT after KA treatment. Our findings indicate that TRIB3 is involved in neuronal apoptosis occurring after KA-induced seizure. The knockdown of TRIB3 effectively protects against neuronal apoptosis *in vitro*, suggesting that TRIB3 may be a potential therapeutic target for the treatment of epilepsy.

## Introduction

Epilepsy is one of the most common neurological disorders in children. More than 70% of patients successfully respond to the therapy with antiepileptic drugs, but a large number of resistant patients continue to experience recurrent seizures, leading to severe incapacitation and cognitive dysfunction (Jensen, 2014). Increasing evidence has revealed that neuronal apoptosis is a prominent feature in epileptogenesis and may contribute to the impairment of cognitive function (Hopkins et al., 2000; Henshall and Simon, 2005). Therefore, the possibility of reducing neuronal apoptosis has potentially important implications in the treatment of epilepsy.

Endoplasmic reticulum (ER) stress can induce neuronal apoptosis in association with many neurological diseases (Imai et al., 2001; Takahashi and Imai, 2003; Rao and Bredesen, 2004; Wang et al., 2005; Han et al., 2015), such as epilepsy, febrile seizures, and Alzheimer’s and Parkinson’s diseases. ER stress occurs under various stressors that provoke the accumulation of unfolded proteins and disturb calcium homeostasis (Rutkowski and Kaufman, 2004; Walter and Ron, 2011). Cells initially respond to ER stress through the activation of the unfolded protein response (UPR) (Walter and Ron, 2011), which in turn elicits an adaptive and restorative effect mainly through the activation of the protein kinase RNA-like ER kinase (PERK), inositol-requiring protein 1α (IRE1α), and transcription factor 6 (ATF6) pathways (Gorman et al., 2012; Sano and Reed, 2013). However, if stressors are persistent or severe, the UPR leads to an execution phase consisting of C/EBP homologous protein (CHOP)-mediated apoptosis (Oyadomari and Mori, 2004; Szegezdi et al., 2006). CHOP is upregulated in human epilepsy and is important for neuronal survival after status epilepticus (SE) (Engel et al., 2013; Sheedy et al., 2014). Although the mechanisms by which CHOP targets apoptosis are not completely understood, it has been shown that ER stress promotes the expression of tribbles pseudokinase 3 (TRIB3), a novel stress-inducible gene induced *via* the ATF4-CHOP pathway, causing cell death (Ohoka et al., 2005).

TRIB3 is a pseudokinase molecule that affects a number of cellular functions (Hegedus et al., 2007; Yokoyama and Nakamura, 2011). TRIB3 has been reported to be highly activated in the presence of a variety of stressors, including the deprivation of neurotrophic factors, hypoxia, and ER stress (Mayumi-Matsuda et al., 1999; Ord et al., 2007; Avery et al., 2010). The induction of TRIB3 can play a detrimental role in the ER stress response of cardiac myocytes by antagonizing cardiac glucose metabolism (Avery et al., 2010), as well as in ER stress-related neuronal apoptosis of PC 12 cells (Zou et al., 2009). TRIB3 is elevated and mediates cell death in Parkinson’s disease (Aime et al., 2015). TRIB3 has also been reported to be an important regulatory protein involved in insulin resistance and tumorigenesis through interfering with AKT activation (Du et al., 2003; Prudente et al., 2012; Salazar et al., 2015a). However, the role of TRIB3 in epilepsy and epilepsy-related brain injury remains controversial. Here, we sought to clarify the role of TRIB3 in neuronal apoptosis mediated by ER stress after seizures. KA activates excitatory glutamate receptors and triggers a delayed type of excitotoxic cell death in various brain regions, including hippocampus, cerebral cortex, and amygdala, which is recognized as an important underlying mechanism in neurodegenerative disorders, such as epilepsy (Wang et al., 2005; Sokka et al., 2007). The seizures in pediatric patients arise frequently in the neocortical structures, which are different from the hippocampal part often seen in adult epilepsy (Wong and Yamada, 2001). In the present study, we used a kainic acid (KA) (10 mg/kg)-induced rat seizure model to investigate the role of TRIB3 and the relationship between TRIB3 and AKT in childhood epilepsy-related neuronal apoptosis of the cortex.

## Materials and Methods

### Experimental Model of SE

Three-week-old male Sprague-Dawley rats (n = 120) were obtained from the Laboratory Animal Center. Care and experimental protocols used in this study were approved by the Animal Research Ethics Committee of Peking University First Hospital. All efforts were made to minimize the number of animals used and their suffering. The experimental animals were randomly divided into the normal control group (n = 60) and the epileptic model group (n = 60). Each group was randomly divided into the following subgroups: 6, 12, 24, and 72 h subgroups (*n* = 15 per subgroup). Rats in the epileptic model group were systemically administered KA (10 mg/kg, i.p., Sigma, USA) (Vincent and Mulle, 2009). Then, animal behavior was observed over a period of 3 h. Rats exhibited standing and jumping behavior, such as wet dog shakes similar to limbic seizures, followed by tonic and generalized seizures observed at 30 min to 3 h after KA application. Onset of SE was determined by the presence of stage 3 to 5 level seizures according to Racine’s scale (Racine, 1972). In the present study, only one rat died after KA application, and 83.3% of the KA-injected rats developed SE. Rats in the control group were injected i.p. with the same volume of physiological saline and divided into the same four time-point subgroups. The animals were euthanized at various time points after KA application. One set of rats (*n* = 6 per subgroup) was perfused through the heart with 0.9% saline to remove blood components for Western blot analysis. The second set (*n* = 6 per subgroup) was perfused transcardially with phosphate-buffered saline (PBS) followed by the 4% paraformaldehyde (PFA) for 30 min, and then brain samples of rats were collected and fixed in 4% PFA for 20 h, transferred to 20% sucrose in PBS overnight at 4°C following the fixation, the brains were subsequently kept in 30% sucrose in PBS until they sank 3 or 4 days later, frozen in liquid nitrogen, and stored at −70°C for further analysis. Fixed brains were ultimately sliced into 10-μm thick sections using a freezing microtome for TUNEL assay and immunofluorescence staining. The brain of rats in the third set (*n* = 3 per subgroup) was used for electronic microscopy, and the rats were transcardially perfused with 0.9% saline followed by 3% PFA and 1% glutaraldehyde in PBS.

### Neuronal Cultures and Transfection of siRNA

Cortical neurons were prepared from embryonic gestation day 18 rat embryos and cultured for 4 days in Neurobasal medium with B27-supplement (GIBCO) on dishes coated with poly-ornithine (Sigma). For the transfection experiments, neurons were transfected with 75 pmol siRNA (TRIB3 or non-targeting control) using Lipofectamine 3000 (Life Technologies) according to the manufacturer’s protocol. At 48 h after transfection, KA (Sigma) was added to the cells to a final concentration of 100 μΜ for 24 h. Then, the neurons were subjected to the TUNEL assay, immunofluorescence staining, Western blot, and qPCR analysis. The target sequence used for TRIB3 siRNA knockdown was 5′-GAAGAAACCGUUGGAGUUTT-3′ (Biolino). Synthetic siRNA targeting rat TRIB3 (Gene ID: 57761) and non-targeting control siRNA were obtained from Beijing Biolino Inc.

### Electron Microscopy

Cortical tissues were removed, immersed in 3% glutaraldehyde in PBS, and cut into semi-thin sections of approximately 1 mm^3^. Sections were then cut into semi-thin sections of 1-μm and ultrathin sections of 100-nm thickness, all in the coronal plane. The changes of cortex ultrastructure were observed by transmission electronic microscopy (JEM-100CX, JEOL, Japan).

### Tunel Assay

About 10 μm sections of brain tissue were used to evaluate apoptosis, and sections of the temporal lobe of the cortex were selected for examination (Defazio et al., 2015). The number of apoptotic neurons was determined using TUNEL staining with an *in situ* cell death detection kit (Roche Applied Science, Germany) according to the protocol provided by the manufacturer. Nuclear staining with DAPI and apoptotic cells labeled with TUNEL (green) were examined under a fluorescence microscope. Six sections were conducted in each group. In each section, the number of TUNEL-positive cells was counted in six counting frames that were randomly selected on images of 400× magnification.

### Western Blotting

Cortical tissues and cultured neurons were lysed using ice-cold radioimmunoprecipitation assay buffer (RIPA) supplemented with a protease inhibitor mixture. Equal amounts of protein were subjected to SDS-PAGE and blotted onto nitrocellulose membranes (Pall). Subsequently, membranes were first incubated for 1 h in 5% skimmed milk and then overnight at 4°C with the following primary antibodies: anti-GRP78 (1:1000; Sigma, USA), anti-CHOP (1:250; Santa Cruz Biotechnology), anti-TRIB3 (1:500; LifeSpan BioSciences, USA), anti-phosphorylated (p)-AKT (1:500) and anti-AKT (1:500; both from Cell Signaling), anti-caspase-3 (1:1000; Cell Signaling), anti-Bax (1:1000; Abcam), and anti-β-actin (1:1000; Zhong Shan Golden Bridge Biotechnology, China). The following day, after being washed three times (TBST, 10 min each), the filters were incubated with horseradish peroxidase-conjugated secondary antibodies (1:5000; Zhong Shan Golden Bridge Biotechnology, China) for 1 h and washed with TBST three times (10 min each), followed by detection using enhanced chemiluminescence. Quantification was performed using ImageJ software. The relative amount of GRP78, CHOP, TRIB3, and p-AKT in control group was arbitrarily assigned as 1 for comparison.

### Immunofluorescence Staining

Frozen sections were immersed in acetone at 4°C for 20 min. Next, the samples were washed with PBS three times (5 min each) and permeabilized with 0.3% Triton X-100. Then, sections were heated in a microwave oven for 10 min in citrate buffer (0.1 M, pH 6.0) for antigen retrieval. Sections were subsequently washed with PBS and blocked with 3% bovine serum albumin (BSA) and 20% normal goat serum for 30 min. Sections were then co-incubated with mouse anti-CHOP (Santa Cruz) and rabbit anti-TRIB3 (LifeSpan, LS-C1535841) or a mixture of mouse anti-TRIB3 (Santa Cruz, sc-390242) and rabbit anti-phospho-AKT (Ser473) (Cell Signaling) and NeuN (Abcam 104224 and 104225) at 4°C overnight. The next day, following three washes in PBS, sections were co-incubated with Alexa Fluor®594 (goat anti-rabbit IgG, red) and Alexa Fluor®488 (goat anti-mouse IgG, green) secondary antibody (Life Technologies) in the dark for 1 h at 37°C and then washed in PBS three times. Next, sections were counterstained with DAPI. Laser scanning confocal microscopy was performed to examine fluorescence.

*In vitro*, neurons were fixed for 20 min using 4% paraformaldehyde. They were then washed three times in PBS and incubated for 1 h in 5% BSA/0.1% Triton X-100. Then, the samples were incubated with antibodies against active caspase-3 (Abcam) and NeuN (Abcam 104224) or co-incubated with mouse anti-TRIB3 (Santa Cruz, sc-390,242) and rabbit anti-phosphor-AKT (Ser473) (Cell Signaling) and then stained with secondary antibody as above.

### Quantitative PCR

RNA was prepared from cultured neurons and cDNA synthesized using 50 U of SuperScript II reverse transcriptase and components given by the vendor (Invitrogen). Primers used in qPCR included: 5′-CGGAGTCAACGGATTTGGTCGTAT-3′ (sense) and 5′-AGCCTTCTCCATGGTGGTGAAGAC-3′ (antisense) for GAPDH cDNA and 5′-CACATCTCTGGCTGCTTCTG-3′ (sense), and 5′-CAGTTGCCTTGCTCTCGTTC-3′ (antisense) for TRIB3 cDNA. Real-time PCR amplification was performed using a SYBR Green PCR Master Mix Kit (Invitrogen). Cycling conditions included denaturation at 95°C for 10 min, followed by 40 cycles of 95°C for 15 s, and extension at 58°C for 1 min. The relative quantity of mRNA was normalized to GAPDH and calculated using the delta-delta method from threshold cycle numbers. On the basis of exponential amplification of the target gene as well as a calibrator, the amount of amplified cDNA at the threshold cycle was given by 2^−ΔΔCt^.

### Statistical Analyses

Quantitative results are expressed as the mean ± SEM. Statistical comparisons were performed using one-way ANOVA, followed by a post hoc analysis (Bonferroni’s test). For each time point *in vivo*, six rats were used, and the *in vitro* assays were repeated more than three times. A *p* < 0.05 was considered significant.

## Results

### Excitotoxic Neuronal Injury and Apoptosis in Rats With KA-Induced Seizure

**Figure 1:**
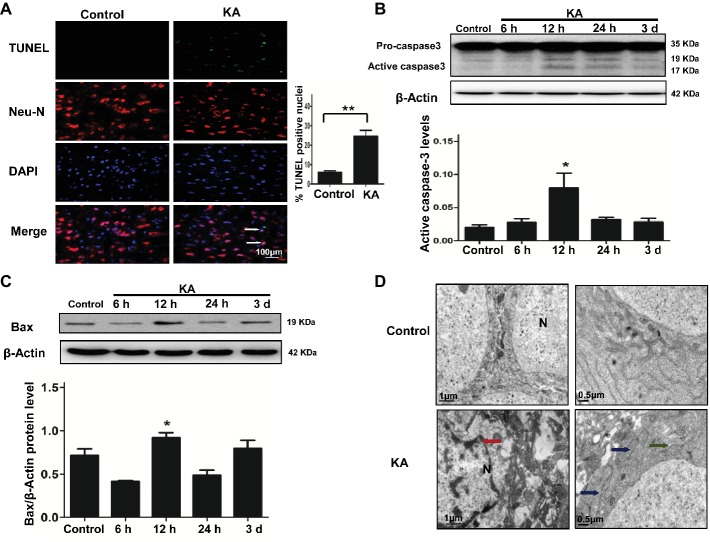
Excitotoxic neuronal cell death is induced by addition of KA. **(A)** TUNEL assay showing apoptotic neurons in rat cortex samples. TUNEL-NeuN-positive cells are indicated by an arrow. Scale bar = 100 μm in all images. Western blot was performed for evaluation of active caspase-3 **(B)** and Bax **(C)** protein levels. *p < 0.05 vs. control (*n* = 6). **(D)** Neuron ultrastructure. Control group: Normal ultrastructure of neurons. KA group: Disrupted or shrunken nuclei (red arrow); swollen mitochondria (blue arrow), with dissolved and ruptured ridges; and dilated rough endoplasmic reticulum (RER) lumens (green narrow). KA, KA group; N, nucleus.

A TUNEL assay and the activation of caspase-3 and Bax were used to assess the level of neuronal injury and apoptosis in the cortex induced by KA. The number of TUNEL-NeuN-positive cells increased by 24 h after KA administration compared with the control group (Figure 1A). Expressions of active caspase-3 and Bax were increased by 12 h post-KA administration and detected by Western blotting (Figures 1B,C). These results suggest that KA administration results in the apoptosis of cortical neurons *in vivo*.

Ultrastructural changes of cortical neurons were observed by electron microscopy. Neurons in the control group showed a normal neuronal structure, including an intact and defined nuclear membrane, normally distributed basic nuclear chromatin, and a normal quantity of organelles. Compared with the changes observed in the control group, striking alterations in ultrastructural changes were manifested in coronal sections of cortical neurons 24 h after KA injection, including nuclear pyknosis and shrinkage of the perikarya, swollen dendrites and mitochondria, rough ER with a swollen lumen, and decreased numbers of ribosomes (Figure 1D).

### ER Stress and the Induction of TRIB3 in Rats With KA-Induced Seizure

**Figure 2:**
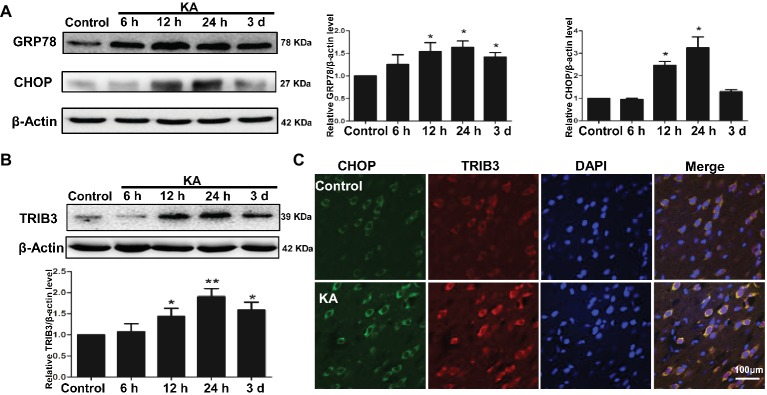
ER stress and the induction of TRIB3 following KA-induced excitotoxicity. Western blots for GRP78 and CHOP **(A)** and TRIB3 **(B)** at various times after KA. **(C)** Double immunofluorescence staining for TRIB3 and CHOP, and the relative amount of GRP78, CHOP, and TRIB3 in the control group was arbitrarily assigned a value of 1 for comparison. *p < 0.05 vs. control (*n* = 6). KA, KA group.

Protein expression of ER chaperones following KA administration was detected by Western blot. Alterations in the ER stress markers GRP78 and CHOP were used to determine whether ER stress occurred following KA-induced seizure. GRP78 was elevated in the rat cortex, beginning at 12 h after KA administration and remained increase until 3 days (Figure 2A). The results also showed that the expression of CHOP in rat cortex increased at 12 h and decreased to control level at 3 days after KA injection. These results indicate that cells within the cortex were undergoing ER stress.

To determine whether TRIB3, a novel ER stress-inducible gene, was involved in neuronal apoptosis, we measured TRIB3 expression by Western blot analysis and then investigated the relationship between CHOP and TRIB3 through double staining. Interestingly, similar to ER stress markers, TRIB3 protein was clearly upregulated by 12 h post-KA and persisted at elevated levels for at least 3 days (Figure 2B). Double immunofluorescence staining for TRIB3 and CHOP showed that the expression levels of TRIB3 and CHOP were increased in cortical neurons 24 h after KA, whereas their expression was barely detectable in the control rat cortex (Figure 2C), similar to our Western blot results.

### TRIB3 Downregulation Reduces KA-Induced Neuronal Apoptosis and ER Stress *in vitro*

**Figure 3:**
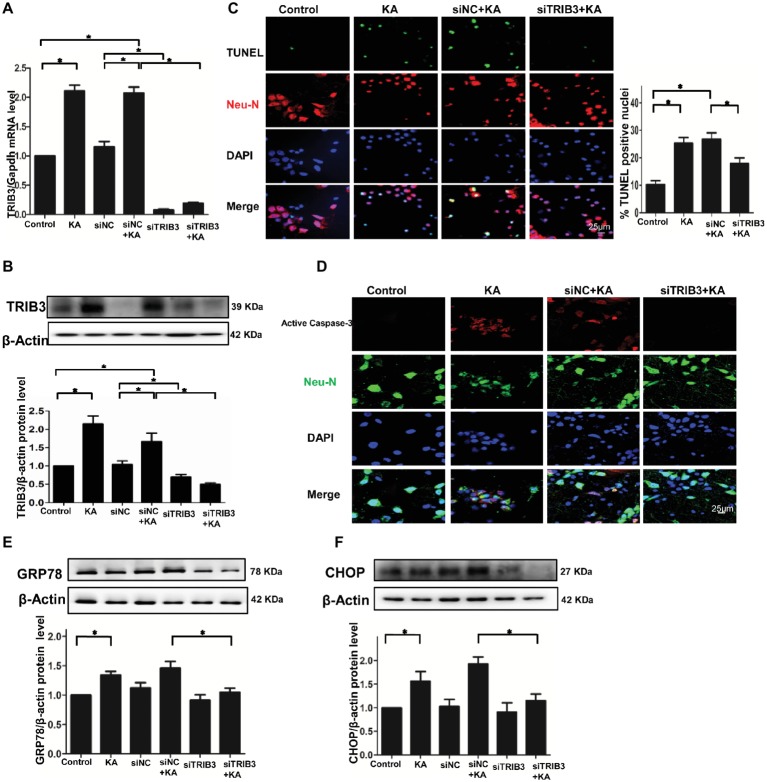
TRIB3 downregulation reduces KA-induced excitotoxic neuronal death *in vitro*. TRIB3 total mRNA **(A)** and protein **(B)** were detected after transfection with siTRIB3. **(C)** Apoptosis of cultured cortical neurons (positive NeuN) was analyzed using the TUNEL assay. **(D)** Activated caspase-3 was detected by immunofluorescence. Expression of GRP78 **(E)** and CHOP **(F)** in cultured neurons was detected by Western blot. *p < 0.05 vs. control. Control, control group; KA, KA-treated group; siNC+KA, siNC+KA-treated group; siTRIB3 + KA, siTRIB3 + KA-treated group.

To explore whether ER stress-induced TRIB3-mediated neuronal apoptosis following KA-induced excitotoxicity, we used three different TRIB3 siRNAs to downregulate TRIB3 in primary cultures of rat cortical neurons treated with or without KA. Downregulation of TRIB3 mRNA and protein was confirmed through qPCR and Western blot analysis, respectively (Figures 3A,B). TUNEL staining revealed an increase in the number of TUNEL-NeuN-positive cells in the KA and siNC+KA group, whereas the targeted downregulation of the TRIB3 (siTRIB3 + KA) group decreased the number of apoptotic cells in cultures compared with the number in the siNC+KA group (Figure 3C). As in the rat cortex, we also detected the activated caspase-3 *in vitro*. Immunofluorescence staining for activated caspase-3 indicated a significant increase in the KA and siNC+KA groups, compared with the level in the untreated control group, and TRIB3 knockdown reduced the activation of caspase-3 caused by KA (Figure 3D).

TRIB3 knockdown has been shown to confer resistance against ER stress in HEK293 and HeLa cells (Ohoka et al., 2005). Therefore, we measured ER stress and TRIB3 expression in KA-treated neuronal cultures. The activation of the ER stress chaperones GRP78 and CHOP was increased following KA exposure, but decreased in the siTRIB3 + KA-treated cells compared with the level in the siNC+KA group (Figures 3E,F).

### TRIB3 Downregulation Inhibits the KA-Mediated Reduction of Phospho-AKT

**Figure 4:**
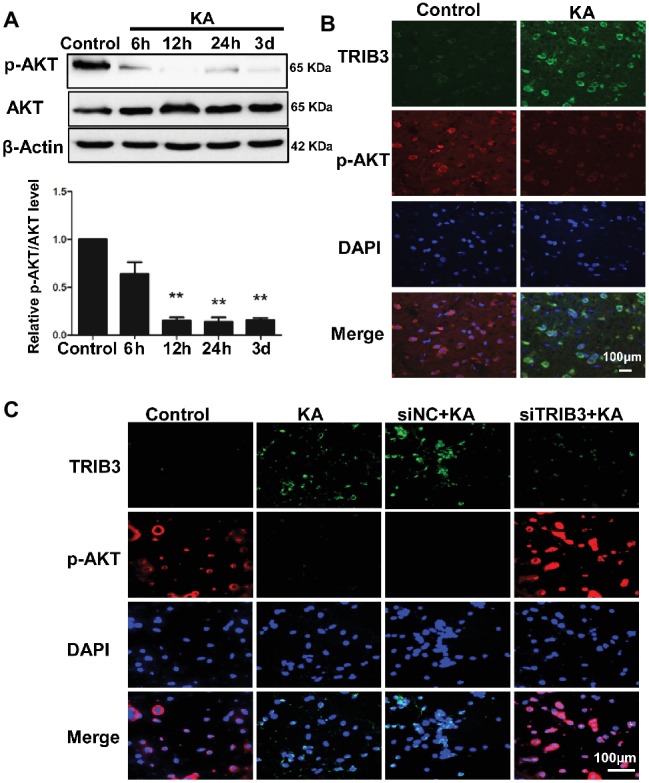
TRIB3 downregulation inhibits the KA-mediated reduction of phospho-AKT. **(A)** Total AKT and phospho-AKT (Ser473) were detected by Western blotting. **(B)** Double immunofluorescence staining for TRIB3 and phospho-AKT. **(C)** Double immunofluorescence staining for TRIB3 and phospho-AKT *in vitro*. **p < 0.05. Control, control group; KA, KA-treated group; siNC+KA, siNC+KA-treated group; siTRIB3 + KA, siTRIB3 + KA-treated group.

Levels of p-AKT (Ser473) were decreased by 12 h following KA administration, whereas total AKT remained unchanged compared with the level in the control (Figure 4A). Double immunofluorescence labeling for TRIB3 and p-AKT in rat cortices isolated at 24 h post-KA revealed that p-AKT was expressed at very low levels in cortical neurons expressing TRIB3. Conversely, in control cortices, TRIB3 expression was seldom observed in neurons immunoreactive for p-AKT (Figure 4B). Therefore, *in vivo*, TRIB3 was upregulated after KA injection, and in the same neurons, p-AKT levels were decreased.

The above results suggest that TRIB3 promotes neuronal apoptosis possibly by interacting with and inhibiting AKT activity in rats with KA-induced seizures. To determine the causal relationship between TRIB3 and p-AKT in excitotoxic neuronal injury, we measured p-AKT (S473) in cultured neurons by immunofluorescence staining. Phosphorylated AKT was rarely detected in KA- and siNC+KA-treated cells, whereas TRIB3 was induced. Importantly, increased p-AKT was observed in the siTRIB3 + KA group (Figure 4C). It is likely that TRIB3 downregulation may at least partially block the decrease in p-AKT caused by KA addition.

## Discussion

In the present study, our data demonstrate that TRIB3 is elevated after KA-induced excitotoxicity *in vivo* and *in vitro*; inhibition of TRIB3 attenuates the neuronal apoptosis observed after kainate *in vitro*; and the targeted downregulation of TRIB3 partially reverses the KA-mediated decrease in phospho-AKT levels *in vitro*. Overall, we show that TRIB3 plays a crucial role in mediating ER stress-induced neuronal apoptosis, possibly by interacting with AKT.

Neuronal apoptosis plays an important role in brain injury after seizures and the formation of chronic epilepsy. Unlike adult epilepsy originating in the hippocampus, in infants and children epilepsy originates in the cerebral cortex (Wong and Yamada, 2001). Neuronal damage involving excitatory glutamate receptors is recognized as an important underlying mechanism in epilepsy; KA-mediated excitotoxicity as a seizure model has been widely used to study the mechanisms of neuronal injury in epileptic disease (Diaz-Ruiz et al., 2013; Liu et al., 2015). ER stress, activation of caspase-3, and changes in the pro-apoptotic protein Bax have been suggested to be involved in neuronal cell death following excitotoxicity induced by KA administration (Sperk et al., 1983; Henshall et al., 2000; Djebaili et al., 2001; Wang et al., 2005; Sokka et al., 2007; Tokuhara et al., 2007; Liu et al., 2009; Urino et al., 2010). A diverse time course of neuronal death has been noted in the KA-lesion rat model, with degenerating neurons being detected at 4 and 6 h, and massive neuronal damage being observed at 1 to 4 days after SE (Hopkins et al., 2000). Understanding the pathological changes in the brain and the mechanism for neuronal damage could further contribute to determining the most suitable time to treat chronic epilepsy. In our study, we used an animal model of childhood epilepsy in which the patients are vulnerable to seizures during the phase of development of the nervous system. We also characterized excitotoxic neuronal injury in the rat cortex following KA administration. We observed the damage of the ultrastructure of the cortex by 24 h after KA injection, as manifested by shrunken nuclei and swollen mitochondria. In addition, the number of dying cells increased by 24 h following KA administration, as determined using the TUNEL assay. Both activation of caspase-3 and protein Bax upregulation occurred in the rat cortex beginning 12 h after KA administration. We also showed that ER stress was involved in excitotoxic neuronal injury induced by KA, as indicated by the upregulation of GRP78 and CHOP. In this study, the ER stress markers GRP78 and CHOP were chosen because they both were involved in each of the three branches of the ER stress pathway (Szegezdi et al., 2006). In addition, the results showed that the increased GRP78 was maintained until 3 days after KA administration, whereas CHOP was significantly decreased at 3 days after KA administration. The study by Sokka et al. showed that CHOP was increased at 24 h and restored to normal level at 48 h in rat after KA administration (Sokka et al., 2007). The study by Irma et al. reported the acute, sub-acute, and chronic changes induced by SE in the immature rat hippocampus. The acute changes included the neuronal death process that mainly occurred within 3 days after SE in the immature rat hippocampus, and then, the activation of inflammatory process and synaptic plasticity triggered the sub-acute changes (Holopainen, 2008). CHOP is known to be involved in ER stress-induced apoptosis (Oyadomari and Mori, 2004), and the expression of CHOP was decreased at 3 days after KA injection, consistent with the change at acute response. The studies by Engel et al. showed that the functions of CHOP in neuronal death induced by seizures might be context-dependent (Engel et al., 2013), and according to Lin’s study, the effects of CHOP may depend on differences in parallel signaling pathways or the duration of activity of individual branches of the unfolded protein response (Lin et al., 2007). Therefore, it might be likely that the different signals activated downstream of ER stress in neuronal damage, neurogenesis, and cellular reorganization in the developing brain after seizures.

Several studies have demonstrated that prolonged ER stress overwhelms UPR survival mechanisms to initiate pro-apoptotic pathways by activating transcription factors (e.g., ATF4 and CHOP), which leads to the overexpression of TRIB3. Furthermore, TRIB3 promotes cellular dysfunction and ultimately results in apoptosis (Ohoka et al., 2005; Bromati et al., 2011; Prudente et al., 2012). ER stress has been shown to be involved in β-cell apoptosis after expression of TRIB3, and TRIB3 is a downstream effector of ER stress and is induced *via* the ATF4-CHOP pathway (Bromati et al., 2011). Cardiomyocyte apoptosis induced by stretching is also mediated by TRIB3 (Cheng et al., 2015). Overexpression of TRIB3 is sufficient to promote neuronal apoptosis even in the presence of nerve growth factor (Zareen et al., 2013) and increased chronic glucose-induced apoptosis in INS-TRIB3 cells (Qian et al., 2008). TRIB3 induction also occurs in response to neurotrophic factor (NGF) deprivation, Parkinson’s disease, and metabolic stress (Mayumi-Matsuda et al., 1999; Du et al., 2003; Hua et al., 2011). However, little information on the effects of TRIB3 in KA-mediated neuronal apoptosis has been previously reported. In the present study, we explored the role of TRIB3 in an epilepsy paradigm. Our data show that TRIB3 is elevated post-KA administration *in vivo* and *in vitro*. Furthermore, TRIB3 upregulation occurs in cortical cells, as observed using immunofluorescence staining following KA administration. *In vitro*, we found that the targeted downregulation of TRIB3 partially reduces the number of apoptotic neurons and decreases the activation of caspase-3 in cortical neurons treated with KA. This suggests that TRIB3 is highly activated in the rat cortex in response to neuronal injury following KA-induced seizure, while TRIB3 mediates neuronal apoptosis and inhibition of TRIB3 protects against neurotoxic injury induced by kainic acid in rats. These findings indicate that TRIB3 could be important for the translation of novel therapeutic approaches in epilepsy.

Under mild or transient ER stress, previous investigators suggested that TRIB3 was primarily regulated by ATF4 and CHOP, and TRIB3 also acted *via* a negative feedback mechanism to inhibit ATF4 and CHOP, thereby promoting cell survival (Rzymski et al., 2008; Su and Kilberg, 2008; Bromati et al., 2011). In the present study, we showed that TRIB3 downregulation attenuated the ER stress response by reducing KA-mediated increase in GRP78 and CHOP. These results suggest that there might be a feedback relationship between TRIB3 and CHOP. TRIB3 plays a crucial role in balancing neuronal survival and death in rats with KA-induced seizures.

Studies have shown that TRIB3 can bind to numerous molecules and regulate their biological functions. Thus, TRIB3 plays a crucial function at the juncture of homeostasis, metabolic disease, and cancer (Humphrey et al., 2010; Hua et al., 2011; Mondal et al., 2016). AKT is known to maintain cell survival by inhibiting apoptosis and promoting cell cycle progression (Song et al., 2005). TRIB3 has been reported to be a negative modulator of AKT (Du et al., 2003) and has been shown to suppress cell survival by interacting with and inhibiting the activation of AKT (Zareen et al., 2013). Loss of TRIB3 enables AKT-driven tumorigenesis *via* forkhead box class O (FoxO) inactivation, and TRIB3 knockdown provides prolonged protection from NGF deprivation, protects axons, and maintains overall neuronal morphology (Zhang et al., 2011; Prudente et al., 2012; Salazar et al., 2015b). Therefore, we explored the level of active AKT [p-AKT (S473)] in the rat cortex. In this study, our data reveal that the levels of active AKT are suppressed in the cortex from 12 h post-KA to 3 days post-KA. Importantly, we found that p-AKT is deficient in cells overproducing TRIB3 in rats following KA-induced seizures. Moreover, we also show that targeted downregulation of TRIB3 partially reverses the decrease in phospho-AKT caused by KA application in cortical cultures, and the deactivation of phospho-AKT corresponds with the induction of TRIB3 in the cortex, supporting the hypothesis that TRIB3 may be responsible for the inhibition of AKT in rats following KA administration. These findings suggest that neuronal apoptosis induced by ER stress is regulated by TRIB3-mediated suppression of AKT in rats with KA-induced seizures. Collectively, epilepsy induces ER stress and overexpression of TRIB3. Additionally, TRIB3 decreases the levels of activated AKT, ultimately resulting in an activation of caspase-3 and upregulation of Bax to mediate neuronal apoptosis. Inhibition of TRIB3 is effective in protecting against neurotoxic injury induced by KA *via* maintaining levels of activated Akt.

Several studies have shown that the expression of TRIB3 and its subcellular localization varied in different tissues, as well as in different disease models. TRIB3 expression was documented in both cytoplasm and nucleus (Prudente et al., 2012; Mondal et al., 2016). In agreement with other researchers, the expression of TRIB3 was observed both in the nucleus and cytoplasm in KA group by using immunofluorescence staining, while the expression of TRIB3 in control group was located in the cytoplasm relatively more than in KA group. Furthermore, it is interesting to explore the mechanisms underlying the changes of intracellular localization of TRIB3 in the KA group.

As shown by our data, targeted downregulation of TRIB3 effectively protects against neuronal injury caused by KA, although the extent of protection is not complete. It is likely that cross-talk with other parallel signaling pathways is fine-tuned at the juncture of neuronal apoptosis in rats with KA-induced seizures. TRIB3 can inhibit AKT phosphorylation and suppress the PI3K/AKT/mTOR pathway (Du et al., 2003), and there is cross-talk between MAPK, NF-κB, and TRIB3 (Okamoto et al., 2007; Rzymski et al., 2008). TRIB3 regulates multiple stress response pathways and fine-tunes stress-inducing and stress-adaptive mechanisms (Mondal et al., 2016). A study by Wei implied that the downregulation of TRIB3 attenuated endoplasmic reticulum stress, enhanced Akt phosphorylation, and protected neuron from apoptosis by stereotaxic injection of TRB3 shRNA lentivirus in the global cerebral ischemia and reperfusion injury in rats (Wei et al., 2017). Erazo’s study showed that the new antitumor drug (ABTL0812) mediated cell death by upregulating the expression of TRIB3 (Erazo et al., 2016). Given the evolving roles and cellular functions of TRIB3 in diseases and biology, it may provide an attractive opportunity for drug design (Eyers et al., 2017). In the present study, we demonstrated the crucial role of TRIB3 in the neuronal apoptosis induced by KA *in vitro*. Further studies would be of help in clarifying the mechanisms for the role of TRIB3 in neuronal apoptosis for a better understanding of the molecular mechanisms underlying epilepsy-related brain damage *in vivo*.

In conclusion, we show that TRIB3 is upregulated in rats with KA-induced seizures and that the induction of TRIB3 and TRIB3-mediated suppression of AKT facilitate neuronal apoptosis in rats following KA-induced seizures. Inhibition of TRIB3 is effective in protecting against neurotoxic injury induced by KA. Our findings suggest that TRIB3 and its regulatory pathways be considered potential and promising therapeutic targets for the treatment of epilepsy.

## Data Availability

All datasets generated for this study are included in the manuscript and/or the supplementary files.

## Ethics Statement

Care and experimental protocols used in this study were approved by the Animal Research Ethics Committee of Peking University First Hospital.

## Author Contributions

JQ and YH conceived and designed the experiments. JZ conducted the experiments. YZ and QL participated in the completion of the experiments. JZ, HJ, and YH analyzed the data. JZ wrote the paper. YH and JQ revised the manuscript. All the authors read and approved the final paper.

### Conflict of Interest Statement

The authors declare that the research was conducted in the absence of any commercial or financial relationships that could be construed as a potential conflict of interest.

## Abbreviations

AKT: protein kinase B
ATF4: transcription factor 4
ATF6: transcription factor 6
CHOP: C/EBP homologous protein
ER stress: endoplasmic reticulum stress
GRP78: glucose-regulated protein 78
KA: kainic acid
PERK: protein kinase RNA-like ER kinase
SE: status epilepticus
TRIB3: tribbles pseudokinase 3
UPR: unfolded protein response.
